# *Yarrowia lipolytica*: a beneficious yeast in biotechnology as a rare opportunistic fungal pathogen: a minireview

**DOI:** 10.1007/s11274-018-2583-8

**Published:** 2018-12-21

**Authors:** Bartłomiej Zieniuk, Agata Fabiszewska

**Affiliations:** Department of Chemistry, Faculty of Food Sciences, Warsaw University of Life Sciences, 159 c Nowoursynowska St., 02-776 Warsaw, Poland

**Keywords:** Drug susceptibility, Fungemia, Opportunistic pathogen, Virulence factors, *Yarrowia lipolytica*

## Abstract

*Yarrowia lipolytica* is one of the most studied “non-conventional” yeast species capable of synthesizing a wide group of valuable metabolites, in particular lipases and other hydrolytic enzymes, microbial oil, citric acid, erythritol and γ-decalactone. Processes based on the yeast have GRAS status (“generally recognized as safe”) given by Food and Drug Administration. The majority of research communications regarding to *Y. lipolytica* claim that the yeast species is non-pathogenic. In spite of that, *Y. lipolytica*, like other fungal species, can cause infections in immunocompromised and critically ill patients. The yeast possess features that facilitate invasion of the host cell (particularly production of hydrolytic enzymes), as well as the protection of the own cells, such as biofilm formation. The aim of this study was to present well-known yeast species *Y. lipolytica* as a rare opportunistic fungal pathogen. Possible pathogenicity and epidemiology of this yeast species were discussed. Antifungal drugs susceptibility and increasing resistance to azoles in *Y. lipolytica* yeasts were also presented.

**Electronic supplementary material:**

The online version of this article (10.1007/s11274-018-2583-8) contains supplementary material, which is available to authorized users.

## Introduction

One of the latest World Health Organization (WHO) report shows a growing threat from antibiotic-resistant microorganisms (WHO report 2017). On the other hand fungal diseases are not less hazardous. The incidence of fungal diseases especially in critically ill patients is thought to be increasing most commonly involving *Candida* and *Aspergillus* species (Beed et al. 2014; Brown et al. 2012). Due to an increase of population of immunocompromised patients and use of antifungal drugs in prophylaxis there have been appeared, other than mentioned above, species of yeasts and molds which can cause severe health problems (Nucci and Marr 2005). The aim of this study was to present well-known yeast species *Yarrowia lipolytica* as a poorly known opportunistic fungal pathogen. Although *Y. lipolytica* has a long history of usage in food industry and an expanding biotechnological potential, the yeast species could be an example of a rare opportunistic fungal pathogen, which cause infections in premature newborns, immunocompromised and critically ill patients (Zhao et al. 2015). Although the first attempt to discuss safety of *Y. lipolytica* usage was made by Groenewald et al. (2014), this review lists possible virulence factors and drug susceptibility in the light of scientific announcements from the last few years, which has not been summarized yet. Moreover, there was exploited the issue by listing all known cases of illness connected with the presence of *Y. lipolytica* yeast what was evidenced in 110 patients.

## *Yarrowia lipolytica* —an overview

*Yarrowia lipolytica* is a member of the Ascomycota phylum. The yeast species was formerly named as *Candida, Endomycopsis* or *Saccharomycopsis lipolytica*. The generic name “*Yarrowia*” refers to David Yarrow, researcher from Delft Microbiology Laboratory (Netherlands), who has identified this genus. The species name “*lipolytica*” is associated with the ability of hydrolyzing lipids (Nicaud 2012). The complete genome of the *Y. lipolytica* E150 strain (CLIB99) was published in 2004 by the Génolevures Consortium. The size of the yeast genome is not constant for all wild and laboratory strains and ranges from 12.7 to 22.1 Mb. The *Y. lipolytica* genome encodes 6448 genes and the number of chromosomes ranges from 4 to 6 (Dujon et al. 2004). Yeast *Y. lipolytica* belongs to heterothallic species with spores of various conjugation types. The frequency of conjugation in natural isolates is very low and wild strains are mostly haploids (Barth and Gaillardin 1997; Kurtzman et al. 2011).

*Yarrowia lipolytica* is widespread in nature. It is isolated from dairy products, such as Camembert and Rokpol cheeses (Roostita and Fleett 1996; Szczepaniak and Wojtatowicz 2011), dry fermented sausages (Flores et al. 2015) and other environments with high content of fats or hydrocarbons, for example rancid margarine, oil-polluted soil and sea water, as the yeast is able to utilize hydrophobic substrates such as hydrocarbons, fatty acids and lipids (Hassanshahian et al. 2012; Krzyczkowska and Fabiszewska 2015). Secretion of lipases by these microorganisms results in lipolysis of triacylglycerols, and proteases hydrolyze proteins. These processes may be desirable in many technological processes, e.g. in creating the characteristic taste and shortening the ripening time, but on the other hand, uncontrolled development of these microorganisms may pose a threat to food quality, e.g. causing an unfavorable appearance and texture (Carreira et al. 1998; Groenewald et al. 2014).

The yeast is used as a model for the study of dimorphism, degradation of hydrophobic substrates, lipid metabolism, protein secretion and peroxisome biogenesis (Beopoulos et al. 2009; Fickers et al. 2005). It is capable of synthesizing a wide group of valuable metabolites, for example lipases, proteases and other hydrolytic enzymes, microbial oil with high content of unsaturated fatty acids, citric acid, erythritol and γ-decalactone (Krzyczkowska 2012; Fabiszewska et al. 2014; Papanikolaou and Aggelis 2011; Tomaszewska et al. 2014). Biotechnological processes based on the yeast species have GRAS (“generally recognized as safe”) status given by Food and Drug Administration (FDA) (Rywińska et al. 2013).

Interestingly, in 2009, the Polish company Skotan SA in cooperation with the Wrocław University of Environmental and Life Sciences, started the production of *Y. lipolytica* biomass (SCP, single cell protein) from waste glycerol and obtained registration of the feed in the European Union. There are also provided the researches on probiotic properties of the species (Rywińska et al. 2013). Only Amano Enzymes uses *Y. lipolytica* lipase in an enzyme preparation under the trade name Amano N-AP. In the past, enzymes from *Y. lipolytica* were used by Fluka, but they ceased their use due to the high thermolability and proteolytic enzyme content in the extract (Brígida et al. 2014). Recently, yeast *Y. lipolytica* can be used for making yeast dough for the feeding of stimulating bee colonies (Apiyarr preparation, Łysoń company, Poland) and the yeast are present in Biopuls preparation for vegetative and generative growth of plants by Micro-life company (Poland) (Londzin et al. 2015; http://www.biopuls.eu/index.php?page=original).

## Epidemiology and pathogenicity of *Yarrowia lipolytica*

First *Y. lipolytica* (*C. lipolytica*) infection was reported in 1976 and it was ocular candidiasis (Nitzulescu and Niculescu 1976). Then in 1985 there was evidenced the first case of fungemia caused by *Y. lipolytica*. It was isolated from blood and intravenous catheter of a patient with recurrent fever (Wehrspann and Füllbrandt 1985). Moreover, Rajagopalan et al. (1996) have reported first occurrence of vaginal colonization by *Y. lipolytica* in asymptomatic 25-year-old woman. Furthermore, case report by Boyd et al. (2017) has shown that *Y. lipolytica* can cause cutaneous infection. 63-year-old immunocompetent woman had epidermal necrosis and minimal dermal inflammation due to yeast presence in her non-healing wound.

Infections due to *Y. lipolytica* have been increasingly described in persons which have been catheterizated for a long time (D’Antonio et al. 2002; Özdemir et al. 2011). Shin et al. (2000) have described a nosocomial outbreak of *Y. lipolytica* fungemia in pediatric patients with central venous catheters. Use of broad-spectrum antibiotics, immunological and hematological disorders, parenteral nutrition and prolonged hospitalization are the main factors affecting development of the infection (Trabelsi et al. 2015). Trabelsi et al. (2015) reported 55 cases of septicemia caused by *Y. lipolytica* occurred between October 2012 and June 2014 in the intensive care unit (ICU) in Tunisian hospital and after treatment 61.8% of patients have been cured. *Y. lipolytica* has been also isolated from nasopharynx, oropharynx, sputum and bronchial washing specimens, as well as from stool samples (Koivikko et al. 1988; Walsh et al. 1989).

Nevertheless, *Y. lipolytica* could be a part of an intestinal mycobiota, because it is isolated also from stool samples of healthy people (Gouba and Drancourt 2015). Irby et al. (2014) have suggested that *Y. lipolytica* should be considered as normal flora of adult respiratory tract. Between 2000 and 2010 *Y. lipolytica* was isolated from 24 patients, which 17 isolates originated from lung tissues.

Supplementary Table 1 summarizes 110 cases of *Y. lipolytica* clinical isolates. The data comes from 22 scientific reports that appeared between 1976 and 2017 (Agarwal et al. 2008; Belet et al. 2006; Blanco et al. 2009; Chang et al. 2001; D’Antonio et al. 2002; Garcia-Martos et al. 1993; Gouba and Drancourt 2015; Irby et al. 2014; Kang et al. 2008; Koivikko et al. 1988; Lai et al. 2012; Levy et al. 2003; Mazumder et al. 2015; Ninin et al. 1997; Nitzulescu and Niculescu 1976; Özdemir et al. 2011; Rajagopalan et al. 1996; Shin et al. 2000; Trabelsi et al. 2015; Walsh et al. 1989; Wehrspann and Fullbrandt 1985; Ye et al. 2011; Zheng et al. 2009). The median age of the patients was 40 (patients were aged from 2 days old to 90 years old). Men accounted for 51.82% of cases (n = 57), woman 22.73% (n = 25), and in other cases there was no information about gender. There were only 6% of known patients under 3 years old (n = 83) and 5% of children between 4 and 10 years old. 31% of patients were between 41 and 60 years old and only 18% were above 60. Yeast isolates were recovered from blood (68.18%, n = 75), lungs (15.45%, n = 17), skin and wounds (5.45%, n = 6), eyes (3.64%, n = 3) and other clinical specimens such as: breast tissue, bronchoalveolar lavage fluid, duodenal mass, intraabdominal abscess, mesenteric mass, sinus aspirate, vagina and stool. When analysing the characteristics of patients in whom *Y. lipolytica* was isolated it can be noticed that usually there were no detected coinfections. In 15 of 29 described cases such coinfections were notified, most often by *C. albicans* (n = 6). Few other *Candida* species (n = 5), staphylococci (n = 4), streptococci (n = 2), *E. coli* (n = 2), *M. tuberculosis* (n = 2) and some other individual species were isolated together with *Y. lipolytica* colonies. In 6 patients more than one species was identified along with *Yarrowia*. There was noticed more than 50 different diseases in people from whom tissues *Y. lipolytica* was isolated. In 24.04% patients (n = 104) factor/disease underlying their weakness and infections was polytraumatism, for 18 patients (17.31%) it was surgery, for 16 patients (15.38%) diabetes, for 7 patients (7.69%) leukemia. If we look at fungal disease which were diagnosed in men from whom *Y. lipolytica* was isolated, the most usually identified disease entity was fungemia (51%) or both: fungemia and catheter-related candidemia (20%). Only one case of granuloma, cutaneous infection, vaginal colonization, ocular candidiasis and acute keratitis were described. Noteworthy, commonly the patients have catheter (88%, n = 82), which was in majority of cases not removed (90%, n = 60). This observations are in line with D’Antonio et al. (2002), Özdemir et al. (2011) and Trabelsi et al. (2015). Predominantly amphotericin B and fluconazole were used to cure the patents, which 42.73% (n = 47) were cleared, and 32 (29.09%) patients died.

## Virulence factors of *Y. lipolytica*

Despite the fact that *Y. lipolytica* is regarded as weakly pathogenic organism, these yeasts present a number of features that allow an effective invasion of the host organism. Hydrolytic enzymes are one of the major factors, which could affect the pathogenicity of *Y. lipolytica*. This fungus is able to produce wide range of hydrolases, such as: proteases, phospholipases and hemolysins, which help in the invasion of tissues (Abbes et al. 2017; Kantarcioglu and Yucel 2002). Phospholipases and proteases secreted by yeasts play an important role in the damage of cell membranes, which consist of lipids and proteins. Moreover, proteases are capable of degrading epithelial and mucosal components, for example collagen and keratin (Fotedar and Al-Hedaithy 2005). Additionally, the most common human fungal pathogen *C. albicans* and other non-*albicans Candida* uses proteolytic enzymes to degradation of antibodies, complement and cytokines (Monod et al. 2002). Similar mechanism may occur in *Y. lipolytica* yeasts, but further studies are needed. *Y. lipolytica* as a former member of the *Candida* genus is compared to *C. albicans*—the most frequently isolated fungal pathogen in humans. Due to high pathogenicity of that yeast species, *C. albicans* is a good example to comparison, despite that these two species are quite evolutionarily distant (Barns et al. 1991).

Kantarcioglu and Yucel (2002) have examined 95 clinical *Candida* isolates and among these isolates four of them were *Y. lipolytica* yeasts. Three of four isolates showed protease activity and none of them showed phospholipase activity. In another research 58 *Y. lipolytica* isolates of blood, urine and vaginal origin were evaluated to produce hydrolytic enzymes in comparison with *C. albicans* and *C. glabrata*. 98.2% of *Y. lipolytica* isolates showed caseinase activity and only vaginal isolates showed phospholipase activity and the hemolytic activity between the three species showed no significant differences (Abbes et al. 2017).

Lipases are hydrolytic enzymes, which may also play its role during infections. Microbial lipases (EC 3.1.1.3) are widely used in food technology, organic chemistry and biotechnology. The lipolytic activity of *Y. lipolytica* for the first time was described by Peters and Nelson (1948), but genes encoding lipase proteins have been discovered since 2000s (Pignède et al. 2000). Lipases and esterases of the *Y. lipolytica* species are coded by the lipase gene family - *LIP* and the major extracellular lipase protein is Lip2p, which is encoded by *LIP2* gene (Fickers et al. 2011). Putative roles of extracellular lipases were discussed by Stehr et al. (2003). The most important function of lipases secreted by microorganisms is lipid digestion and thanks to lipolysis the yeast could use hydrophobic carbon sources for growth. Utilization of unusual carbon sources by *Y. lipolytica* is frequently discussed topic by biotechnologists (Fickers et al. 2005). According to Stehr et al. (2003) free fatty acids and other products of lipolysis (mono- and diacylglycerols) could affect different immune cells, initiate the inflammatory process and support adhesion to host cells.

Holzschu et al. (1979) compared 11 yeasts species of industrial interest with *C. albicans* for their potential pathogenicity. Untreated and cortisone-treated mice were inoculated with yeasts, such as *Y. lipolytica, S. cerevisiae* and other *Candida* species. *C. tropicalis* caused infections similar to *C. albicans*. Other yeasts were not recovered after 6 days or recovered, but did not caused infections. In accordance to *Y. lipolytica*, authors suggested that maximum growth temperature of that yeasts is near 34 °C, therefore the species was not lethal or invasive for mice. Walsh et al. (1989) checked the virulence of fungemia isolate of *Y. lipolytica* in murine model. Mice received different dilutions of yeasts via the lateral tail vein. All animals survived 14 days of experiment. Mouse which received the highest inoculum of *Y. lipolytica* had two abscesses in the left kidney and in the culture with kidney tissue that yeasts were present. Other tissues (brain, liver, spleen) were free of microbial contaminants and lesions. In comparison with *Y. lipolytica, C. albicans* caused mortality in all mice within the 2 week of experiment.

Adhesion to the host cells and hyphae formation can be also other significant virulence factors of the yeast species. *Y. lipolytica* is species of dimorphic yeast, which means that it grows as yeast-like cells or forms hyphae or pseudohyphae (Barth and Gaillardin 1997). Mechanism of dimorphic transition in *Y. lipolytica* is not well characterized, because filamentation of cells is a complex process. Over the years, scientists have published plenty of reports about factors and compounds affecting or inhibiting cells filamentation. It could be induced by carbon and nitrogen source, pH value, temperature, oxygenation and it’s also dependent on strain specificity (Kerkhoven et al. 2016). *CLA4* gene is reported to play role in dimorphism of *Y. lipolytica*. It encodes Cla4 protein kinase, which is highly homologous to Cla4 protein kinases of *C. albicans* and *S. cerevisiae*. Deletion of *CLA4* gene is not lethal, however, due to lack of this gene mutants of *Y. lipolytica* cannot form mycelium (Szabo 2001). Other genes involved in yeast-to-hyphae transition are *YlRAC1* and *YlBMH1*. First of them encoding a G protein of the Rho family and the second encoding a 14-3-3 protein. Transcription levels of these genes increased during the yeast-to-hyphae transition (Hurtado et al. 2000; Hurtado and Rachubinski 2002). Moreover, Morales-Vargas et al. (2012) have reported 61 up- and 165 downregulated genes involved in dimorphism, where some of these genes were homologous with *S. cerevisiae*.

Biofilm is a form of microbial community that is associated with a surface (Desai et al. 2014). Due to presence of extracellular matrix and complex structure, biofilms protect yeast cells against antifungal agents (Fanning and Mitchell 2012). D’Antonio et al. (2002) reported that *Y. lipolytica* produced large amount of slime in glucose-based medium and that confirms the ability of biofilm production and also adhesion to and colonization of plastic central venous catheter. Abbes et al. (2017) studied biofilm formation by *Y. lipolytica* in comparison with *C. albicans* and *C. glabrata*. Biofilms development have been examined in vitro (using 96-well polystyrene microtiter plates) as well as in vivo in a rat subcutaneous model. In contrast to *Candida* species, biofilms obtained by *Y. lipolytica* after catheter subcutaneous implementation seemed to be more compact hyphal layer and also number of cells was significantly greater.

## Drug susceptibility of *Yarrowia lipolytica*

Antifungals are antimycotic drugs used to treat or prevent fungal diseases. These medications can be divided into the following classes: polyenes, azoles, echinocandins, and others. Polyenes, such as Amphotericin B and Nystatin bind to sterols in the cell membrane, mainly ergosterol, which causes disruption of the cell membrane integrity and resulting in leakage of ions and other cytoplasmic components and cell lysis (Odds et al. 2003). Azoles are the largest group of antifungal agents. The most common in clinical use are triazoles, such as Fluconazole and Itraconazole. Mechanism of their action is to inhibit 14a-demethylation of lanosterol in the ergosterol biosynthetic pathway (Ghannoum and Rice 1999). Echinocandins (Anidulafungin, Caspofungin and Micafungin) are inhibitors of glucan synthesis in the cell wall (Ghannoum and Rice 1999). Another used antifungal drug is Flucytosine, also known as 5-fluorocytosine. Flucytosine is converted inside fungal cell to 5-fluorouracil and other compounds, resulting in inhibition of DNA synthesis (Odds et al. 2003).

Antifungal susceptibility testing of the yeasts provides meaningful information for epidemiological, clinical and therapeutic issues. As mentioned before, *Y. lipolytica* was often isolated from patients with central venous catheters. In some cases, removal of central vein catheter in conjunction with an antifungal drug was the best way in treatment of catheter-related candidemia. On the other hand, sometimes catheter could not be removed due to patient’s condition or type of catheter. Antifungal-lock technique (ALT) was proposed as an alternative method for treatment of catheter-related infections. ALT consists of filling the catheter lumen with antimicrobials and leaving them for an appropriate period of time (Mermel et al. 2001). Özdemir et al. (2011) reported first case of using ALT in combination with systemic therapy to treat *Y. lipolytica* fungemia. Intravenous caspofungin and caspofungin-lock therapy was initiated in 9-year-old patient with neuroblastoma. Solution of antifungal, dextrose and heparin was placed in the lines for 12 h. Cultures were negative after 4 days of treatment, and therapy was stopped after 14 days and no relapse was seen then.

A panel of experts of the European Society of Clinical Microbiology and Infectious Diseases (ESCMID) and the European Confederation of Medical Mycology (ECMM) presented an overview of data on rare invasive yeast infections. *Y. lipolytica* was also mentioned in the report and authors claimed that clinical significance of that yeast species is uncertain and minimal inhibitory concentration (MIC) of fluconazole is higher than MIC obtained for *C. albicans* (Arendrup et al. 2014). MIC distribution of *Yarrowia lipolytica* isolates is presented in Table 1. The data comes from seven scientific reports (Blanco et al. 2009; D’Antonio et al. 2002; Diekema et al. 2009; Lai et al. 2012; Shin et al. 2000; Walsh et al. 1989; Zhao et al. 2015). Additionally, MIC_50_ and MIC_90_ values were calculated, which represents MIC values at which 50% or 90% of isolates in population are inhibited, respectively. Both MIC, MIC_50_ and MIC_90_ are important parameters in describing the susceptibility of isolates to antimicrobials.

**Table 1:** MIC distribution of *Yarrowia lipolytica* isolates

Antifungal classes	Antifungal agent	Number of isolates with MIC (µg/ml) of														MIC_50_*	MIC_90_*	Observations
		0.008	0.016	0.032	0.064	0.125	0.25	0.5	1	2	4	8	16	32	≥ 64			
Polyene antifungals	Amphotericin B						7	22	19	6	4	1				1	2	59
Azole antifungals	Ketoconazole		1		3	3	4									–	–	11
	Fluconazole						1	2	3	14	15	1	2	5	8	4	> 64	51
	Itraconazole	1			2	1	7	16	3	1			3			0.5	2	34
	Posaconazole			1		3	3	12	5	4	1	3				0.5	4	32
	Voriconazole	1	1	9	9	5	2		4	4						0.064	2	35
Echinocandins	Anidulafungin			3		7	5	6	2	1						–	–	24
	Caspofungin		1		1	7	12	9	1							0.25	0.5	31
	Micafungin			1		2	8	8	5							–	–	24
Others	5-Fluorocytosine					1		3			4	3	6			–	–	17

*MIC50 and MIC90 values were calculated for antifungals with at least 30 observations

Zhao et al. (2015) compared susceptibility of 14 *Y. lipolytica* isolates to 9 different antifungal drugs. All 14 isolates showed low MICs to echinocandins and amphotericin B, 4 isolates had MICs of < 4 µg/ml to flucytosine and susceptibility to azoles was more diverse, where MIC values for fluconazole were highest. Diekema et al. (2009) examined the susceptibility of 658 clinical isolates of rare species of *Candida* yeasts (including 16 strains of *Y. lipolytica*) to amphotericin, fluconazole, posaconazole, voriconazole, anidulafungin, caspofungin and micafungin. Echinocandins and voriconazole exhibited highest activity against *Y. lipolytica*, and MICs of that antifungals were not higher than 2 µg/ml, but most of the tested strains were not susceptible to amphotericin B and fluconazole.

Barchiesi et al. (1999) compared in vitro activity of five antifungals (fluconazole, itraconazole, ketoconazole, flucytosine and amphotericin B) against uncommon clinical isolates of *Candida* spp. Results showed that strains differ in susceptibility to antifungal agents. Among the tested strains authors identified a strain of *Y. lipolytica*, with low susceptible to fluconazole and also that strain was cross-resistant to ketoconazole and itraconazole. Sixty-seven percent of *Y. lipolytica* strains have been defined as isolates with reduced susceptibility to flucytosine, therefore that antifungal agent is rarely used in monotherapy of fungal infections.

The studies quoted above show that voriconazole, caspofungin, micafungin and anidulafungin may be better treatment options than fluconazole or 5-fluorocytosine. Results show also a phenomenon of increasing resistance to azoles. Mechanism of azole resistance occurring in *Candida* genus is associated with, among others, overexpression of *CDR1* (*Candida* drug resistance gene), *CDR2* (a homologue of *CDR1*) and *MDR1* (multidrug resistance gene) genes, which encode efflux pumps, that are capable of transporting antifungal drugs out of the cell. Another mechanism is occurring a point mutations in genes encoding enzymes, which should be targeted by antifungal drug. Mutations in *ERG11* gene (encoding lanosterol 14α-demethylase) prevent from biding azoles, resulting in a lack of inhibition of encoded enzyme, which is essential for ergosterol biosynthesis (major sterol found in cell membranes of fungi). Moreover, mutations can lead to overexpression of Erg11 protein, which increase resistance to azoles (Gołąbek et al. 2015; Whaley et al. 2017). Additionally, mutations in *ERG3* gene (encoding Δ^5,6^-desaturase) can lead to accumulation of 14a-methylfecosterol in fungal membrane instead of ergosterol. As a result of reducing the amount of ergosterol in the membrane, resistance to azoles and polyenes increases (Kanafani and Perfect 2008). In the case of echinocandins resistance in *C. albicans* and other *Candida* spp., it results from point mutations in the *FKS1* gene (involved in β-1,3-d-glucan synthesis). Substitution of amino acids in protein lead to elevating MIC value for echinocandins 5- to 100-fold (Cowen et al. 2015). Yeast can also be resistant to 5-fluorocytosine. This is due to the fact that mutations in cytosine deaminase and uracil phosphoribosyltransferase lead to defects in flucytosine metabolism what result in resistance to that antifungal drug (Ghannoum and Rice 1999). Mechanisms of resistance in *Y. lipolytica* have not been described yet, however, those listed above may also occur in this yeast species.

## Conclusion

*Yarrowia lipolytica* is an yeast species phylogenetically distant from *S. cerevisiae* or other well-studied yeast species and known from its interesting physiological features. It is considered as a non-pathogenic microorganism and the U.S. Food and Drug Administration has given its metabolites the GRAS status. Moreover, the species is an inseparable element of the microflora accompanying many food products with a long history of occurrence in human diet. Nevertheless, in the light of the reviewed cases of yeast infections, *Y. lipolytica* may be also considered as a rare opportunistic fungal pathogen in patients with compromised immunity and those with catheters. Infections caused by this yeast species are very rare, however *Y. lipolytica* might be an epidemiological problem for critically ill patients in the future due to increasing resistance to antifungal drugs. The reported in the literature strains of *Y. lipolytica* differed in susceptibility to antifungal agents, mainly fluconazole and other azoles were investigated. In some cases mentioned in the review, infections were resolved without treatment, which may suggest low virulence of the species, therefore further studies are needed to compare the virulence of *Y. lipolytica* with other *Candida* spp. and to confirm its pathogenicity, as most of the yeast occurrences in patients were reported not earlier than 20 years ago. An interesting issue are virulence factors of *Y. lipolytica*, which have been proposed in the review. With a lot of certainty occurrence of *Y. lipolytica* infections yet do not cross with its usage in various industries as a microorganism with a huge biotechnological potential, but the pathogenicity of each foodborne microorganism should be monitored up to date.

## Electronic supplementary material

Below is the link to the electronic supplementary material.

Supplementary material 1 (PDF 485 KB)
