# Supplementary material for: Yarrowia lipolytica: a beneficious yeast in biotechnology as a rare opportunistic fungal pathogen: a minireview
Source: World J Microbiol Biotechnol. 2018 Dec 21;35(1):10. doi: 10.1007/s11274-018-2583-8 (PMC6302869; doi:10.1007/s11274-018-2583-8)
Supplement: Supplementary file 1 — Supplementary material 1 (PDF 485 KB) [file 11274_2018_2583_MOESM1_ESM.pdf]

Supplementary Table 1. Characteristics of patients in whom *Yarrowia lipolytica* was isolated*.

No.	Clinical origin	Sex	Age	Fungal disease	Underlying factors/diseases	Coinfections	CT	CT removal	Outcome	Additional information	Antibiotic therapy**	Reference
1.	Blood culture	F	57 yo	Fungemia Catheter-related candidemia	Recurrent stomach ulcers, stroke, cerebral atrophy, fever, alcohol abuse	*C. albicans* in urine	+	+	Cleared	-	KTC	[Wehrspann & Fullbrandt, 1985]
2.	Blood culture	M	61 yo	Fungemia Catheter-related candidemia	Progressive dyspnea, cough, fever, pneumonitis	*C. albicans*	+	+	Cleared	-	FLC, then MFG	[Lai et al., 2012]
3.	Blood culture	F	15 yo	Fungemia	Acute myelogenous leukemia	*Stenotrophomonas maltophilia*	+	+	Cleared	-	FLU, ISP, AMP	[Chang et al., 2001]
4.	Blood culture	M	2 yo	Fungemia Catheter-related candidemia	Fever, tubercular meningitis	*M. tuberculosis*	+	+	ND	-	INH, RIF, PZA, STR, AMB	[Agarwal et al., 2008]
5.	Blood culture	F	18 yo	Fungemia Catheter-related candidemia	Acute lymphoblastic leukemia	-	+	+	Died	Patient received allogeneic bone marrow transplantation	SXT, AMB, ACV	[D'Antonio et al., 2002]
6.	Blood culture	M	9 yo	Fungemia Catheter-related candidemia	Neuroblastoma, fever	-	+	+	Cleared	Patient received autologous stem cell transplantation	SCF, AMK, then MEM, TEC, FLC, and subsequently CAS	[Ozdemir et al., 2011]
7.	Blood culture	M	1 mo	Fungemia	Necrotizing enterocolitis, malabsorption	-	+	+	Cleared	-	FLC	[Shin et al., 2000]
8.	Blood culture	M	2 mo	Fungemia	Bacterial infection (meningitis)	group b streptococci	-	NA	Cleared	-	Broad-spectrum antibiotics	[Shin et al., 2000]
9.	Blood culture	F	8 yo	Fungemia	Acute myelogenous leukemia, chemotherapy	-	+	-	Cleared	-	FLC, then AMB	[Shin et al., 2000]
10.	Blood culture	M	14 yo	Fungemia	Acute myelogenous leukemia, chemotherapy	-	+	-	Cleared	-	FLC, then AMB	[Shin et al., 2000]
11.	Blood culture	M	4 yo	Fungemia	Aplastic anemia, immunotherapy	-	+	+	Cleared	-	FLC	[Shin et al., 2000]

12.	Blood culture	M	54 yo	Fungemia Catheter-related candidemia	Suppurative thrombophlebitis, alcohol abuse, cholecystectomy for cholelithiasis and cholecystitis	-	+	+	Cleared	-	Six different antibacterial agents in different combinations	[Walsh et al., 1989]
13.	Blood culture	M	46 yo	Fungemia	Enlarged liver, alcohol abuse	-	-	NA	Died	-	ND	[García-Martos et al., 1993]
14.	Blood culture	F	40 yo	Fungemia Catheter-related candidemia	Thrombocythemia, tuberculosis,	*C. albicans*, *C. glabrata*, *C. guilliermondii* and *Geotrichum* sp.	+	+	Cleared	Patient received allogeneic bone marrow transplantation	AMB	[Ninin et al., 1997]
15.	Blood culture	ND	12 yo	Fungemia Catheter-related candidemia	Cystic pancreatic fibrosis	*C. glabrata*	+	+	Cleared	-	CAZ, GEN, then MEM, and AMK, and subsequently change to VRC	[Blanco et al., 2009]
16.	Blood culture	F	86 yo	Fungemia Catheter-related candidemia	Recurrent urinary tract infections and vesical neoformation with peritoneal fibrosis, bilateral hydronephrosis	*Escherichia coli, Klebsiella pneumoniae, Enterobacter cloacae, Pseudomonas aeruginosa, A. baumannii, C. albicans, C. glabrata* and *C. tropicalis*	+	+	Cleared	-	CAS	[Blanco et al., 2009]
17.	Blood culture	F	90 yo	Fungemia	General weakness and hydronephrosis, diarrhea symptoms	*E. coli* and methicillin-resistant coagulase-negative staphylococcus	+	+	Died	Infection after raw beef ingestion	Antibiotic treatment and also FLC and then AMB	[Kang et al., 2008]
18.	Blood culture	M	13 yo	Fungemia Catheter-related candidemia	Acute Lymphoblastic Leukemia	-	+	+	Cleared	-	CFP, VAN, FLC	[Ye et al., 2011]

19.	Blood culture	M	2 do	Fungemia Catheter-related candidemia	Intestinal obstruction and suspected sepsis	*C. albicans*	+	+	Cleared	-	CTX and FLC, then AMB, and subsequently AMB + CAS	[Belet et al., 2006]
20.	Blood culture	F	4 mo	Fungemia Catheter-related candidemia	Gastroesophageal reflux	methicillin-resistant *S. aureus*, *C. albicans*	+	+	Cleared	-	VAN, broad-spectrum antimicrobials and FLC, then AMB + CAS	[Belet et al., 2006]
21.	Blood culture	M	21 yo	Fungemia Catheter-related candidemia	Polytraumatism, Abdominal surgery	ND	+	+	Cleared	-	Broad-spectrum antibiotics, AMB, FLC	[Trabelsi et al., 2015]
22.	Blood culture	M	39 yo	Fungemia	Polytraumatism, Cerebral surgery	ND	+	+	Cleared	-	Broad-spectrum antibiotics	[Trabelsi et al., 2015]
23.	Blood culture	F	60 yo	Fungemia	Ischemic cerebrovascular accident, Diabetes	ND	+	+	Died	-	Broad-spectrum antibiotics, FLC, AMB	[Trabelsi et al., 2015]
24.	Blood culture	F	48 yo	Fungemia	Polytraumatism, Cerebral surgery	ND	+	+	Cleared	-	Broad-spectrum antibiotics, FLC	[Trabelsi et al., 2015]
25.	Blood culture	F	32 yo	Fungemia	Status epilepticus, Diabetes	ND	+	-	Died	-	Broad-spectrum antibiotics, FLC, AMB	[Trabelsi et al., 2015]
26.	Blood culture	F	15 yo	Fungemia	Menometrorrhagia, Acute anemia, Abdominal surgery	ND	+	+	Died	-	Broad-spectrum antibiotics, FLC	[Trabelsi et al., 2015]
27.	Blood culture	M	78 yo	Fungemia	Chronic obstructive pulmonary disease	ND	+	-	Cleared	-	Broad-spectrum antibiotics, FLC	[Trabelsi et al., 2015]

28.	Blood culture	M	50 yo	Fungemia	Rectosigmoid tumor, Abdominal surgery	ND	+	-	Died	-	Broad-spectrum antibiotics, FLC, AMB	[Trabelsi et al., 2015]
29.	Blood culture	M	60 yo	Fungemia	Chronic renal failure, Diabetes	ND	+	-	Died	-	Broad-spectrum antibiotics, AMB	[Trabelsi et al., 2015]
30.	Blood culture	M	47 yo	Fungemia	Polytraumatism, Abdominal surgery	ND	+	+	Cleared	-	Broad-spectrum antibiotics, AMB	[Trabelsi et al., 2015]
31.	Blood culture	M	27 yo	Fungemia	Thoracic traumatism, Abdominal surgery	ND	+	+	Cleared	-	Broad-spectrum antibiotics, FLC	[Trabelsi et al., 2015]
32.	Blood culture	M	43 yo	Fungemia	Polytraumatism	ND	+	+	Cleared	-	Broad-spectrum antibiotics, FLC	[Trabelsi et al., 2015]
33.	Blood culture	M	52 yo	Fungemia	Renal failure, Cerebrovascular accident	ND	+	+	Died	-	Broad-spectrum antibiotics, AMB, FLC	[Trabelsi et al., 2015]
34.	Blood culture	M	68 yo	Fungemia	Chronic obstructive pulmonary disease	ND	-	NA	Cleared	-	Broad-spectrum antibiotics, FLC	[Trabelsi et al., 2015]
35.	Blood culture	M	36 yo	Fungemia	Polytraumatism, Multiple surgery	ND	+	+	Died	-	Broad-spectrum antibiotics, AMB	[Trabelsi et al., 2015]
36.	Blood culture	M	18 yo	Fungemia	Polytraumatism	ND	+	+	Cleared	-	Broad-spectrum antibiotics, AMB	[Trabelsi et al., 2015]

37.	Blood culture	M	51 yo	Fungemia	Polytraumatism	ND	+	+	Cleared	-	Broad-spectrum antibiotics, FLC, AMB	[Trabelsi et al., 2015]
38.	Blood culture	M	46 yo	Fungemia Catheter-related candidemia	Polytraumatism, Multiple surgery	ND	+	+	Cleared	-	Broad-spectrum antibiotics, AMB	[Trabelsi et al., 2015]
39.	Blood culture	F	62 yo	Fungemia	Acute pancreatitis	ND	+	ND	Died	-	Broad-spectrum antibiotics, AMB	[Trabelsi et al., 2015]
40.	Blood culture	M	20 yo	Fungemia	Polytraumatism	ND	+	+	Died	-	Broad-spectrum antibiotics, FLC	[Trabelsi et al., 2015]
41.	Blood culture	M	73 yo	Fungemia	Myocardial infarction, Renal failure, Diabetes	ND	+	ND	Died	-	Broad-spectrum antibiotics	[Trabelsi et al., 2015]
42.	Blood culture	M	74 yo	Fungemia	Polytraumatism, Diabetes	ND	+	+	Died	-	Broad-spectrum antibiotics, FLC, AMB	[Trabelsi et al., 2015]
43.	Blood culture	F	14 yo	Fungemia	Status epilepticus	ND	+	+	Cleared	-	Broad-spectrum antibiotics, FLC	[Trabelsi et al., 2015]
44.	Blood culture	M	57 yo	Fungemia	Polytraumatism, Diabetes	ND	+	+	Died	-	Broad-spectrum antibiotics	[Trabelsi et al., 2015]
45.	Blood culture	F	35 yo	Fungemia Catheter-related candidemia	Post-operative shock, Surgery - cesarian	ND	+	+	Cleared	-	Broad-spectrum antibiotics, FLC	[Trabelsi et al., 2015]
46.	Blood culture	M	16 yo	Fungemia	Polytraumatism, Multiple surgery	ND	+	+	Cleared	-	Broad-spectrum antibiotics, FLC, AMB	[Trabelsi et al., 2015]

47.	Blood culture	F	58 yo	Fungemia	Diabetes, Heart failure, Cardiomyopathy, Surgery	ND	+	+	Died	-	Broad-spectrum antibiotics, FLC, AMB	[Trabelsi et al., 2015]
48.	Blood culture	M	50 yo	Fungemia	Guillain-Barre syndrome, Pneumopathy	ND	+	+	Died	-	Broad-spectrum antibiotics	[Trabelsi et al., 2015]
49.	Blood culture	M	36 yo	Fungemia	Polytraumatism	ND	+	ND	Cleared	-	Broad-spectrum antibiotics, AMB	[Trabelsi et al., 2015]
50.	Blood culture	F	45 yo	Fungemia Catheter-related candidemia	Pneumonia	ND	+	+	Died	-	Broad-spectrum antibiotics, AMB	[Trabelsi et al., 2015]
51.	Blood culture	M	21 yo	Fungemia	Polytraumatism	ND	+	ND	Cleared	-	Broad-spectrum antibiotics, AMB	[Trabelsi et al., 2015]
52.	Blood culture	M	61 yo	Fungemia Catheter-related candidemia	Polytraumatism	ND	+	+	Cleared	-	Broad-spectrum antibiotics, AMB	[Trabelsi et al., 2015]
53.	Blood culture	M	26 yo	Fungemia	Polytraumatism	ND	+	+	Cleared	-	Broad-spectrum antibiotics	[Trabelsi et al., 2015]
54.	Blood culture	M	78 yo	Fungemia	Polytraumatism, Abdominal surgery	ND	+	ND	Died	-	Broad-spectrum antibiotics	[Trabelsi et al., 2015]
55.	Blood culture	M	27 yo	Fungemia	Polytraumatism	ND	+	+	Cleared	-	Broad-spectrum antibiotics, AMB	[Trabelsi et al., 2015]
56.	Blood culture	M	83 yo	Fungemia Catheter-related candidemia	Thoracic traumatism, Heart failure	ND	+	ND	Died	-	Broad-spectrum antibiotics	[Trabelsi et al., 2015]

57.	Blood culture	M	42 yo	Fungemia	Polytraumatism, Diabetes	ND	+	+	Cleared	-	Broad-spectrum antibiotics, AMB	[Trabelsi et al., 2015]
58.	Blood culture	M	30 yo	Fungemia Catheter-related candidemia	Polytraumatism, Diabetes	ND	+	+	Cleared	-	Broad-spectrum antibiotics	[Trabelsi et al., 2015]
59.	Blood culture	M	45 yo	Fungemia	Polytraumatism, Diabetes	ND	+	ND	Died	-	Broad-spectrum antibiotics, FLC, AMB	[Trabelsi et al., 2015]
60.	Blood culture	M	50 yo	Fungemia	Pharyngeal cancer	ND	-	-	Died	-	Broad-spectrum antibiotics, AMB	[Trabelsi et al., 2015]
61.	Blood culture	M	61 yo	Fungemia	Acute colitis, Diabetes, Abdominal surgery	ND	+	ND	Cleared	-	Broad-spectrum antibiotics, FLC	[Trabelsi et al., 2015]
62.	Blood culture	M	52 yo	Fungemia	Hemorrhagic rectocolitis, Abdominal surgery	ND	+	ND	Died	-	Broad-spectrum antibiotics, AMB	[Trabelsi et al., 2015]
63.	Blood culture	F	36 yo	Fungemia	Chronic renal failure, Diabetes	ND	+	+	Cleared	-	Broad-spectrum antibiotics	[Trabelsi et al., 2015]
64.	Blood culture	F	25 yo	Fungemia	Diabetes, Epilepsy, Caustic oesophagitis	ND	+	+	Died	-	Broad-spectrum antibiotics, AMB	[Trabelsi et al., 2015]
65.	Blood culture	M	38 yo	Fungemia	Thoracic and abdominal trauma, Diabetes	ND	+	+	Died	-	Broad-spectrum antibiotics, AMB	[Trabelsi et al., 2015]
66.	Blood culture	M	29 yo	Fungemia	Abdominal trauma, Abdominal Surgery	ND	+	ND	Died	-	Broad-spectrum antibiotics, AMB, FLC	[Trabelsi et al., 2015]

67.	Blood culture	M	30 yo	Fungemia Catheter-related candidemia	Thoracic trauma, Surgery	ND	+	+	Died	-	Broad-spectrum antibiotics	[Trabelsi et al., 2015]
68.	Blood culture	M	46 yo	Fungemia	Diabetes, Bronchopneumopathy	ND	+	ND	Died	-	Broad-spectrum antibiotics, FLC	[Trabelsi et al., 2015]
69.	Blood culture	M	32 yo	Fungemia	Nosocomial pneumopathy	ND	+	+	ND	-	Broad-spectrum antibiotics, AMB	[Trabelsi et al., 2015]
70.	Blood culture	M	34 yo	Fungemia	Polytraumatism	ND	+	ND	Cleared	-	Broad-spectrum antibiotics, FLC	[Trabelsi et al., 2015]
71.	Blood culture	M	64 yo	Fungemia	Hemorrhagic, Cerebrovascular accident, Cerebral surgery	ND	+	+	Died	-	Broad-spectrum antibiotics, AMB, FLC	[Trabelsi et al., 2015]
72.	Blood culture	M	60 yo	Fungemia	Acute pulmonary edema, Diabetes	ND	+	+	Cleared	-	Broad-spectrum antibiotics, FLC	[Trabelsi et al., 2015]
73.	Blood culture	M	4 yo	Fungemia	Soda intoxication, Caustic oesophagitis	ND	+	+	Died	-	Broad-spectrum antibiotics	[Trabelsi et al., 2015]
74.	Blood culture	M	40 yo	Fungemia	Polytraumatism	ND	+	+	Died	-	Broad-spectrum antibiotics	[Trabelsi et al., 2015]
75.	Blood culture	M	18 yo	Fungemia	Polytraumatism	ND	+	+	Cleared	-	Broad-spectrum antibiotics, AMB	[Trabelsi et al., 2015]
76.	Breast tissue	ND	ND	Postivie culture for *Y. lipolytica*	Breast carcinoma	ND	ND	ND	ND	-	-	[Irby et al., 2014]

77.	Bronchoalveolar lavage fluid	ND	ND	Postivie culture for *Y. lipolytica*	Breast carcinoma	ND	ND	ND	ND	-	-	[Irby et al., 2014]
78.	Duodenal mass	ND	ND	Postivie culture for *Y. lipolytica*	Benign granuloma	ND	ND	ND	ND	-	-	[Irby et al., 2014]
79.	Eye	ND	ND	Ocular candidiasis	ND	ND	ND	ND	ND	-	ND	[Nitzulescu & Niculescu, 1976]
80.	Eye	ND	ND	Ocular candidiasis	ND	ND	ND	ND	ND	-	ND	[Nitzulescu & Niculescu, 1976]
81.	Eye	ND	ND	Ocular candidiasis	ND	ND	ND	ND	ND	-	ND	[Nitzulescu & Niculescu, 1976]
82.	Eye	F	57 yo	Acute keratitis	Corneal ulcer with persistent epidefect of the left eye	-	-	-	Cleared	-	NAT, FML	[Lai et al., 2012]
83.	Intraabdominal abscess	F	42 yo	Fungemia	Liver transplantation	*Enterococcus faecium*, viridans group streptococci, methicillin-resistant *S. epidermidis*,	+	+	Died	-	DAP, DOR, MFG, and then AMB	[Mazumder et al., 2015]
84.	Lung	ND	ND	Postivie culture for *Y. lipolytica*	Melanoma	ND	ND	ND	ND	-	-	[Irby et al., 2014]
85.	Lung	ND	ND	Postivie culture for *Y. lipolytica*	Benign granuloma	ND	ND	ND	ND	-	-	[Irby et al., 2014]
86.	Lung	ND	ND	Postivie culture for *Y. lipolytica*	Necrotizing granuloma	ND	ND	ND	ND	-	-	[Irby et al., 2014]
87.	Lung	ND	ND	Postivie culture for *Y. lipolytica*	Benign granuloma	ND	ND	ND	ND	-	-	[Irby et al., 2014]
88.	Lung	ND	ND	Postivie culture for *Y. lipolytica*	Non-small-cell lung carcinoma	ND	ND	ND	ND	-	-	[Irby et al., 2014]
89.	Lung	ND	ND	Postivie culture for *Y. lipolytica*	Cryptogenic organizing pneumonia	ND	ND	ND	ND	-	-	[Irby et al., 2014]
90.	Lung	ND	ND	Postivie culture for *Y. lipolytica*	Benign granuloma	ND	ND	ND	ND	-	-	[Irby et al., 2014]
91.	Lung	ND	ND	Postivie culture for *Y. lipolytica*	Synovial sarcoma	ND	ND	ND	ND	-	-	[Irby et al., 2014]

92.	Lung	ND	ND	Postivie culture for *Y. lipolytica*	Renal cell carcinoma, Benign granuloma	ND	ND	ND	ND	-	-	[Irby et al., 2014]
93.	Lung	ND	ND	Postivie culture for *Y. lipolytica*	Benign granuloma	ND	ND	ND	ND	-	-	[Irby et al., 2014]
94.	Lung	ND	ND	Postivie culture for *Y. lipolytica*	Benign granuloma	ND	ND	ND	ND	-	-	[Irby et al., 2014]
95.	Lung	ND	ND	Postivie culture for *Y. lipolytica*	None	*M. tuberculosis*	ND	ND	ND	-	-	[Irby et al., 2014]
96.	Lung	ND	ND	Postivie culture for *Y. lipolytica*	None	ND	ND	ND	ND	-	-	[Irby et al., 2014]
97.	Lung	ND	ND	Postivie culture for *Y. lipolytica*	Non-small-cell lung carcinoma	ND	ND	ND	ND	-	-	[Irby et al., 2014]
98.	Lung	ND	ND	Postivie culture for *Y. lipolytica*	Chronic myelogenous leukemia, Pulmonary alveolar proteinosis	ND	ND	ND	ND	-	-	[Irby et al., 2014]
99.	Lung	ND	ND	Postivie culture for *Y. lipolytica*	Melanoma	ND	ND	ND	ND	-	-	[Irby et al., 2014]
100.	Lung	ND	ND	Postivie culture for *Y. lipolytica*	None	ND	ND	ND	ND	-	-	[Irby et al., 2014]
101.	Mesenteri c mass	ND	ND	Postivie culture for *Y. lipolytica*	Benign cystic mass	ND	ND	ND	ND	-	-	[Irby et al., 2014]
102.	Sinus aspirate	F	46 yo	Localized infection	Chronic sinusitis	*S. aureus*, *P. aeruginosa*	-	-	Cleared	-	KTC	[Walsh et al., 1989]
103.	Skin	F	63 yo	Cutaneous infection	Chronic obstructive pulmonary disease and hypertension	-	-	-	Cleared	-	CIP, ITC, then FLC	[Boyd et al., 2017]
104.	Skin	M	39 yo	Granuloma	Surgical operation, therapies including antibiotics, anti-tuberculosis agents, prednisone and cyclophosphamide	-	-	-	ND	-	ND	[Zheng et al., 2009]
105.	Skin	ND	ND	Postivie culture for *Y. lipolytica*	Meningioma	ND	ND	ND	ND	-	-	[Irby et al., 2014]
106.	Skin	ND	ND	Postivie culture for *Y. lipolytica*	Acute myelogenous leukemia	ND	ND	ND	ND	-	-	[Irby et al., 2014]
107.	Stool	ND	ND	Postivie culture for *Y. lipolytica*	Acute myelogenous leukemia	ND	ND	ND	ND	-	-	[Irby et al., 2014]
108.	Ulceration wound	F	46 yo	Soft-tissue infection	Adrenal insufficiency and a chronic ulcer lesion over the right knee	-	-	-	Cleared after surgery	-	-	[Lai et al., 2012]

109.	Vagina	F	25 yo	Vaginal colonisation	Cervicitis with squamous metaplasia	-	-	-	Cleared	-	-	[Rajagopalan et al., 1996]
110.	Wound	M	73 yo	Postivie culture for *Y. lipolytica*	The patient was bitten by rats/Necrosis of the left temporalis area and the left ear	*Mucor* sp.	ND	ND	ND	The patient was transferred to hospice care	CAZ, OXA, MTZ, AMB	[Levy et al., 2003]

*ND - No Data; NA - Not Applicable; yo - years old; mo - months old; do - days old; + (Yes); - (No); F – Female; M – Male; CT – Catheter

**Antimicrobial agents abbreviations: ACV – Acyclovir, AMB – Amphotericin B, AMK – Amikacin, AMP – Ampicillin, CAS – Caspofungin, CAZ – Ceftazidime, CFP – Cefoperazone, CIP – Ciprofloxacin, CTX – Cefotaxime, DAP – Daptomycin, DOR – Doripenem, FLC – Fluconazole, FLU – Flucloxacillin, FML – Fluorometholone, GEN – Gentamicin, INH – Isoniazid, ISP – Isepamicin, ITC – Itraconazole, KTC – Ketoconazole, MEM – Meropenem, MFG – Micafungin, MTZ – Metronidazole, NAT – Natamycin, OXA – Oxacillin, PZA – Pyrazinamide, RIF – Rifampicin, SCF – Cefoperazone-Sulbactam, STR – Streptomycin, SXT – Trimethoprim-Sulfamethoxazole, TEC – Teicoplanin, VAN – Vancomycin, VRC – Voriconazole
